# Low-Cost GNSS Receivers for Local Monitoring: Experimental Simulation, and Analysis of Displacements

**DOI:** 10.3390/s16122140

**Published:** 2016-12-15

**Authors:** Ludovico Biagi, Florin Cătălin Grec, Marco Negretti

**Affiliations:** Department of Civil and Environmental Engineering, Politecnico di Milano, Como 22100, Italy; florincatalin.grec@polimi.it (F.C.G.); marco.negretti@polimi.it (M.N.)

**Keywords:** low-cost GNSS receivers, u-blox, landslide monitoring, static surveying, time series analysis, displacements analysis

## Abstract

The geodetic monitoring of local displacements and deformations is often needed for civil engineering structures and natural phenomena like, for example, landslides. A local permanent GNSS (Global Navigation Satellite Systems) network can be installed: receiver positions in the interest area are estimated and monitored with respect to reference stations. Usually, GNSS geodetic receivers are adopted and provide results with accuracies at the millimeter level: however, they are very expensive and the initial cost and the risk of damage and loss can discourage this approach. In this paper the accuracy and the reliability of low-cost u-blox GNSS receivers are experimentally investigated for local monitoring. Two experiments are analyzed. In the first, a baseline (65 m long) between one geodetic reference receiver and one u-blox is continuously observed for one week: the data are processed by hourly sessions and the results provide comparisons between two processing packages and a preliminary accuracy assessment. Then, a network composed of one geodetic and two u-blox receivers is set up. One u-blox is installed on a device (slide) that allows to apply controlled displacements. The geodetic and the other u-blox (at about 130 m) act as references. The experiment lasts about two weeks. The data are again processed by hourly sessions. The estimated displacements of the u-blox on the slide are analyzed and compared with the imposed displacements. All of the results are encouraging: in the first experiment the standard deviations of the residuals are smaller than 5 mm both in the horizontal and vertical; in the second, they are slightly worse but still satisfactory (5 mm in the horizontal and 13 mm in vertical) and the imposed displacements are almost correctly identified.

## 1. Introduction

The purpose of local monitoring is to closely model displacements, evolution and deformations of civil engineering structures and local natural phenomena, such as landslides; in this paper, we will focus on landslides, but the proposed methodology and results can be extended to many other applications of local monitoring. A landslide is a serious geologic hazard defined as the movement of a mass of rock, debris, or earth down a slope [1]. In Italy, landslides kill or injure people almost every year. A recently published Italian catalogue covers a 68-years window, from 1941 to 2010, and lists 3139 landslide events that have resulted in deaths, missing persons, and injured people [2]. Globally, these geologic hazards are causing destructions that can be estimated to billions of dollars every year. Due to this, and because of the high number of human casualties, an accurate and continuous monitoring of areas prone to landslides is desired.

Landslides monitoring is a multi-disciplinary approach which leads to the estimation of the slope movement and the design and planning of safety measures. Landslides can move slowly (millimeter or centimeter per year) over an extended period of time. Then, for various reasons, they can disastrously accelerate and collapse. Their monitoring must allow an accurate modeling of the phenomenon both in space and in time; moreover the latency of the results should be as small as possible.

In the past, landslides were monitored only by geotechnical instruments such as accelerometers and displacement transducers. In the last fifty years, techniques that integrated geodetic and geotechnical measurements have been developed and extensively applied, with satisfactory results: more details are given in [3,4]. One of the best examples is the case of Meirato landslide (Italy), where total stations, together with other geodetic instruments, were used with success [5]. Moreover, in the last twenty years, a new technique emerged as an alternative to traditional approaches: landslides monitoring by Global Navigation Satellite System (GNSS) technology [6,7].

Typically, the relative static processing is applied. It exploits data from (at least) two GNSS receivers, simultaneously tracking the same satellites. One receiver is the reference station, the other is called the rover. By double differencing the carrier phase observations, the baseline between the reference and the rover is estimated. In local monitoring, one or more reference receivers are installed in stable locations outside the landslide and several rovers, whose positions are estimated and monitored in time, are installed on the landslide.

An early study on this technique was conducted in 2000 by Gili et al. [8]. Their research was focused on the landslide of Vallcebre (Spain) and they compared the results of single frequency GNSS receivers with the results from photogrammetry and geodetic measurements. It was found out that the precision of GNSS is anywhere between 12 and 16 mm in the horizontal plane and 18–24 mm in elevation. With the availability of dual frequency receivers, the results improved and horizontal precisions better than 1 cm were constantly observed [9,10,11].

In [12,13], a procedure to process regional permanent networks is described, implemented, and tested. The procedure allows to completely automate all the process: the quality check of the data, their processing and the network adjustment, the quality check of the results and the analysis of the time series of the estimated coordinates. Such an approach, clearly adapted to the specific application, can be set up also for local permanent networks and can provide an automated monitoring of the displacements in quasi-real-time, for example, by hourly sessions.

In landslides monitoring, the high cost of geodetic receivers (several thousands of USD) represents a problem at least at two levels: firstly, the initial setup of the control network is very expensive, especially in case many monitoring sites are needed on the landslide; secondly, in the case of a landslide event, the receivers can be damaged or lost forever, and this means a significant budget deficit. This clearly could discourage this monitoring technique in many cases. Nowadays, GNSS receivers exist that cost few hundreds of USD and output single frequency phase observations: the aim of this paper is to evaluate their performances in local monitoring applications.

Some of the early works in this direction involved monitoring of the Aggenalm landslide situated in the Bavarian Alps, Germany. In general, the final accuracy of the solution is influenced by baseline length, satellite visibility, nearby environment and can be even better than 1 cm in perfect working conditions [14]. Furthermore, two experiments carried out in the Alps, provided a first, satisfactory guess of the accuracy of low-cost, single frequency receivers in landslide monitoring [15]. A recent research is presented also in [16]: in this case, the accuracy of low-cost receivers is assessed for local monitoring by processing daily sessions: accordingly to the baseline length, the final accuracy range from 1 mm (very short baseline) to 1 cm (30 km long baseline).

The present research continues the experimentation of low-cost receivers in local monitoring. The first objective is to test their accuracy by processing short (e.g., hourly) sessions, that provide a quasi-real-time monitoring. Secondly, their reliability in detecting displacements at the sub centimeter level will be tested. This implies two features:

(1) the capability to correctly detect displacements occurrence, without false alarms (significance); and
(2) the ability to correctly identify at least the order of magnitude of a displacement (congruence).

In monitoring, a possible approach is the adoption of a two-level monitoring network: geodetic receivers are used as reference stations while low-cost receivers are installed on the monitored area. This is already cost effective, because one reference station could suffice and it does not risk any damage or loss. The other possible scheme implies the installation of low-cost receivers both in reference and in monitored sites and is even more cost effective. Therefore, a comparison between these two alternatives is interesting.

In general, scientific packages provide the best accuracies in GNSS data processing because they implement the state of the art in modeling data and relevant physical phenomena. However, scientific packages require highly specialized and well trained users because they allow a wide selection of processing parameters. On the contrary, several commercial packages exist that implement more simplified models but are certainly more easy to use: at least at the local scale, it is interesting to check their results. A comparison between scientific and commercial packages is then useful.

We presented a first experimental test in [17]: a horizontally-controlled movement was imposed to a low-cost rover, and the estimated displacements were compared with the imposed ones. These results were very promising. Therefore, we want to complete this first experiment with a longer dataset and checking displacements both in horizontal and vertical directions: rigorous significance and congruence tests will be applied to address the above questions, following a methodology firstly presented in [18].

Firstly, we analyze a week of acquisitions on a single baseline: this is referred to as Milano Test and is aimed at a preliminary assessment of accuracy and comparisons between GNSS processing packages.

Then, a simulation of a local network for displacements monitoring is set up. One geodetic and two u-blox receivers are used. One u-blox acts as rover and is installed on a device (slide) that allows to apply controlled displacements: in this way, the rover is gradually moved from the initial position at given intervals. The geodetic and the other u-blox are at about 130 m from the rover and act as reference. The experiment lasts about two weeks. The rover data are processed with respect to the two reference stations by hourly sessions. The estimated displacements are analyzed and compared with the imposed ones: the analysis is completed by significance and congruence tests. This is referred to as the Como Test.

Section 2 presents the low-cost receivers adopted in the experiments. Section 3 discusses the results of Milano test. Section 4 discusses the results of Como test. Section 5 presents future work plans and Section 6 summarizes the conclusions.

## 2. Adopted Low-Cost Receivers and Software

The following problems of low-cost receivers, not experienced by geodetic receivers, must be mentioned. Firstly, they use narrow-band filters to limit the sampling frequency that could cause noisier measurements. Additionally, their antennas do not have the multipath mitigation techniques that are present in geodetic antennas, such as vision correlators from Novatel [19] or the a posteriori multipath estimator (APME) technique from Septentrio (Leuven, Belgium) [20]: this could cause significant problems in locations with reflection sources, and can lead to errors above the accepted threshold for monitoring applications [21]. For high accuracy applications, the antenna calibration and its phase center variations (PCV) are needed [22,23]: in particular, the vertical estimates are clearly affected by the accuracy of available PCV. However, available PCVs from International GNSS Service do not include corrections for antennas of low-cost GPS receivers. All of these shortcomings could cause noisier time series of estimated coordinates, especially in height.

In both experiments, u-blox receivers [24] are used: u-blox is a Swiss-based company (Thalwil) and a leading provider of wireless and positioning sensors and modules for the automotive, industrial, and consumer markets.

As previously stated, the comparison of two GNSS processing packages was a secondary task: the two chosen programs were Bernese GPS Software Version 5.2 (BSW5.2) from the Astronomical Institute of University of Bern (Switzerland) and Leica Geo Office Version 8.3 (LGO) from Leica Geosystems (Heerbrugg, Switzerland).

BSW5.2 [25,26] is one of the leading software packages for the international geodetic community. To optimally tune the processing options, the users should possess very good theoretical knowledge and experience in GNSS data processing. BSW5.2 was used as benchmark, to compare the results of LGO. The main processing parameters are reported in Table 1. LGO is a commercial package that supports many surveying sensors and techniques, for example, GNSS, terrestrial positioning systems, and leveling data [27]. Where a choice is possible, the adopted processing parameters in LGO (Table 1) were identical to those of BSW5.2: in LGO, the ambiguity resolution algorithm is fixed and cannot be chosen by the user.

Usually, a time window of approximately 15–60 min is considered for local monitoring applications in quasi-real-time [28,29]: we decided to process hourly sessions and data acquired at 1 Hz. The session length and the adopted sampling rate allow a good number of available observations under any conditions. At the same time, in the most of the monitoring applications, an hourly processing guarantees an acceptable latency of the results.

## 3. Milano Test Processing and Analysis

The data of the Milano Test were acquired and courteously provided by Riccardo Barzaghi and Mirko Reguzzoni, Department of Civil and Environmental Engineering, Politecnico di Milano. The dataset is relevant to a short baseline (approximately 65 m long) formed by the permanent station (Topcon Odyssey RS) of Politecnico di Milano—Milano Leonardo Campus and one u-blox LEA-4T. Both receivers were mounted on the roof of a building owned by Politecnico, at about 24 m above ground level: the acquisition site was already used in several previous geodetic tests and offers an optimal observation environment. The survey lasted one week, from 14 to 20 March 2014 (respectively, days of the year—DOY 073 and 079). Our analysis of Milano data is mainly aimed at a first assessment of potential accuracies reached by low-cost receivers.

Firstly, we processed the whole week with BSW5.2 to estimate a reference position of the rover. Then, the hourly sessions were processed to estimate the rover positions: the time series of the residuals relative to the reference position were finally computed:

(1) $$\delta x_{i}=x_{i}-\bar{x}$$

where $\bar{x}$ is the reference position of the rover, $x_{i}$ is the estimated position for session *i*, and $\delta x_{i}$ is the vector of the residuals. The residuals are presented as local east, north, and up with respect to the reference position.

In total, 168 results were obtained. Five (3%) were clearly blundered: Table 2 presents their three dimensional (3D) residuals.

The statistics for the other sessions are presented in Table 3. Overall, the results are completely satisfactory: the root mean square error (RMSE) of the solutions is 2.1 mm for east, 4.9 mm for north, and 4.5 mm for up directions.

The same hourly sessions were processed with LGO. In this case, four blunders were identified: for these solutions, ambiguities were not resolved at all and led to large errors, with the maximum reaching 1.30 m (Table 4). In Table 5 the basic statistics of the other sessions are reported.

BSW5.2 and LGO results are similar. No bias exists between the two packages. LGO’s RMSE are slightly better in horizontal while vertical results of the two packages are similar. As expected, the vertical statistics are the worst: in this case, beside the usual reasons (satellite geometry), this is probably caused also by the lack of antenna PCVs for u-blox. For a more detailed comparison between BSW5.2 and LGO it was decided to count the sessions with absolute errors in the following classes: 0–5 mm, 5–15 mm, 15–30 mm and 30–50 mm (Table 6).

The results are satisfactory for monitoring applications. All of the residuals are smaller than 50 mm: the horizontal errors are well below 0.5 cm in most of the cases, especially for east with residuals under 5 mm for all sessions processed in LGO, and almost all sessions (99.4%) processed in BSW5.2 (see columns East in Table 6) As expected, vertical residuals have worse statistics compared to the horizontal residuals, but are still satisfactory: 74.2% of the BSW5.2 results present residuals smaller than 5 mm, and the remaining 25.8% are between 5 and 15 mm. For LGO, 69.7% of the vertical residuals are smaller than 5 mm, and the remaining 30.3% are between 5 and 15 mm (see columns Up in Table 6). Moreover, in the processing of our dataset, a commercial and user-friendly software, like LGO, provides results that are completely consistent with those of BSW5.2, with a slight degradation in the height estimates. In Table 6 it can be seen that both horizontal and vertical residuals do not pass 15 mm for almost all sessions in the case of both software. There are only four exceptions: for BSW5.2 one session (0.6%) for east and two sessions for north, and for LGO only one session for north.

**Figure 1:**
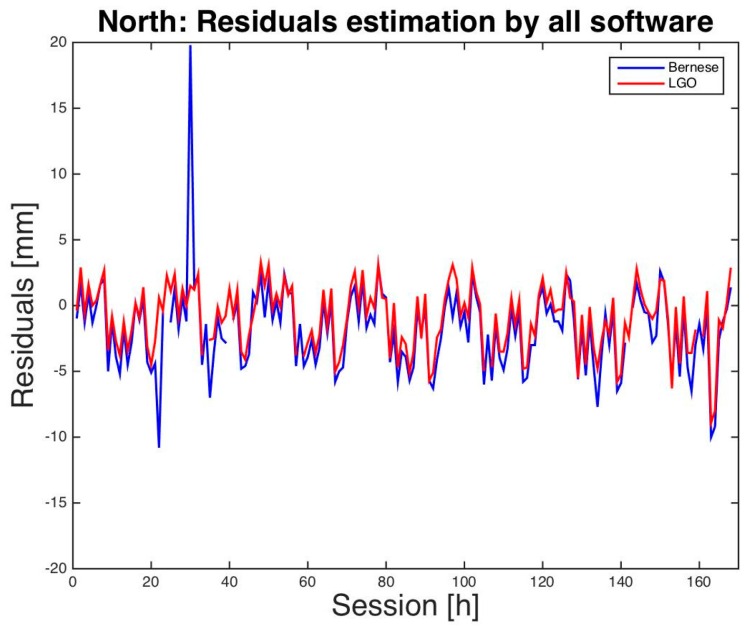
Milano test. North residuals of hourly sessions. BSW5.2 in blue, LGO in red.

**Figure 2:**
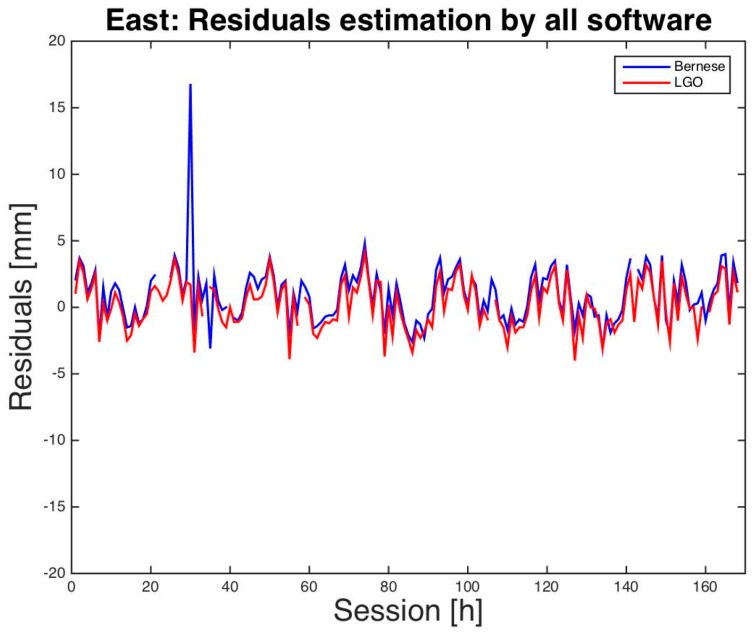
Milano test. East residuals of hourly sessions. BSW5.2 in blue, LGO in red.

**Figure 3:**
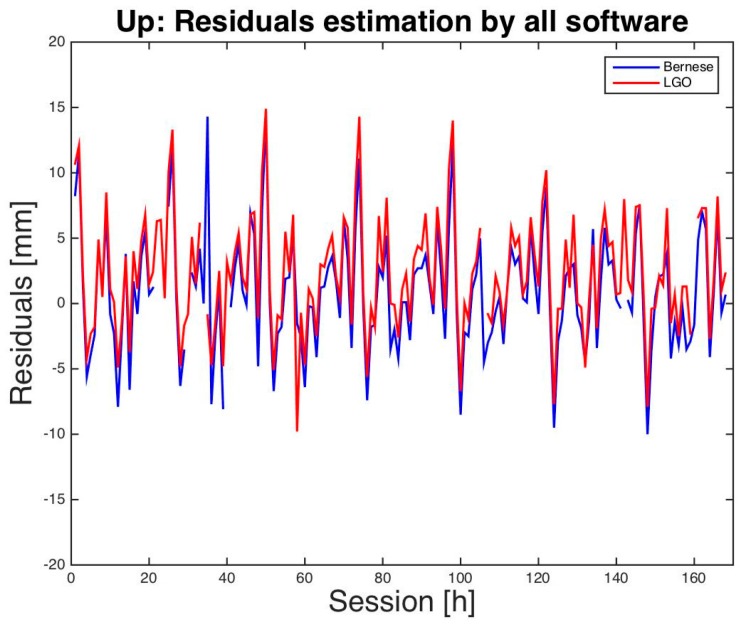
Milano test. Vertical residuals of hourly sessions. BSW5.2 in blue, LGO in red.

Finally, a graphical comparison between LGO and BSW5.2 residuals is presented in Figure 1, Figure 2 and Figure 3. Just for a better visual inspection, three residuals that exceed the interval [−2.0 cm, +2.0 cm] have been removed from the graphs: two for BSW5.2 and one for LGO. Almost all the other residuals show similar magnitude and sign for almost all the sessions and a daily period appears, in particular for the height: this is probably caused by multipath interference, particularly significant for low-cost antennas.

In conclusion, we had five (BSW, Table 2) plus four (LGO, Table 4) blundered results, which seems to be a relatively big number. These blunders are mainly caused by occasional problems in the data but probably also because of another reason: packages for post-processing of GNSS static sessions are optimized for data acquired by geodetic receivers and not for low-cost receivers. Other, and more detailed, results relevant to the Milano test are discussed in [30].

## 4. The Como Test

The test was set up to achieve the main research objectives. The test was carried out on the Como Campus of Politecnico di Milano: it was performed in 2016, between DOYs 28 and 42 (28 January–11 February).

### 4.1. The Surveying

**Figure 4:**
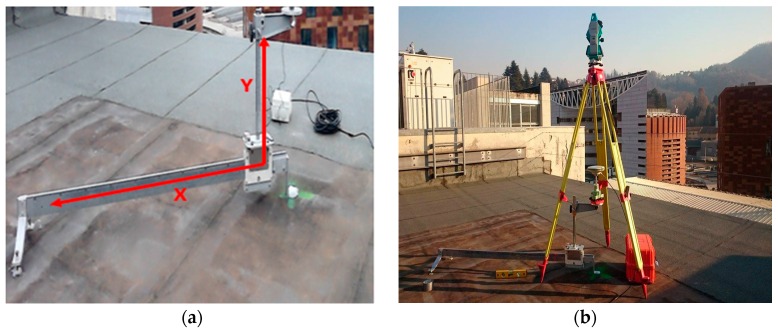
The slide of the Como Test. (**a**) The device: *X* displacement 1.3 m, *Y* displacement 0.3 m; and (**b**) the total station used to orient the horizontal *X* axis exactly to the northeast (azimuth of 45° from north) direction.

To impose displacements, a device (slide) that allowed controlled horizontal and vertical displacements was used (Figure 4). It was designed and built at Department of Environment, Land and Infrastructure Engineering, Politecnico di Torino. It is composed of a calibrated hardened steel bar (*X* axis), with a special support for a GNSS antenna on a vertical steel bar (*Y* axis). When the slide is perfectly leveled, the *X* axis materializes as a horizontal direction while the *Y* axis is the vertical direction.

The movements of the antenna support are controlled by a wheel and pivot activated manually. Two millimetric tapes allow the user to control the imposed displacements for *X* and *Y* axes. The system guarantees the check of the displacements with a precision better than 1 mm, which is certainly sufficient for our test. In total, horizontal displacements up to 1.30 m and vertical displacements up to 30 cm can be applied. The slide was fixed on the roof of the building in Castelnuovo street. The *X* axis of the slide was oriented clockwise 45° from the north direction with the help of a total station. In this way, horizontal displacements are equally distributed in the north and east directions.

In total, three receivers were used:

- The Como European Permanent Network (EPN) station [31]. The station consists in a Topcon Odyssey RS receiver and its antenna mounted on a pillar on the roof of the building in Valleggio street: the building, with its height of 30 m, is the dominant structure inside the campus and provides optimal open sky conditions in all directions. This was used as the primary reference station in the data processing scheme.
- One u-blox NEO-7P receiver. This antenna was installed on a point with well-known coordinates, at about 3 m from the Como EPN station. This was used as the secondary reference station in the estimation of the rover.
- One u-blox NEO-7P receiver which simulated a monitored rover. It was installed on the slide, at about 130 m in the northwest direction with respect to the reference stations on the roof of a building (Castelnuovo street), about 10 m in height.

NEO-7P is a consumer-grade GNSS receiver equipped with u-blox next generation GPS platform NEO-7P and an active GPS antenna [32]. The main technical characteristics of u-blox NEO-7P are:

- high-precision GPS < 1 m, in the configuration satellite-based augmentation system (SBAS) + precise point positioning (PPP),
- differential GPS by SBAS or Radio Technical Commission for Maritime Service (RTCM),
- raw measurement data (GPS),
- 56 channels: GPS L1 C/A; GLONASS L1 FDMA; QZSS L1 C/A; SBAS,
- GNSS receiver: GPS, GLONASS, quasi-zenith satellite system (QZSS), and Galileo,
- antenna gain: 27 dB.

The rover u-blox used its default antenna. The secondary reference u-blox was paired with a Tallysman TW3150 GPS L1 single frequency antenna: it is still a low-cost device as its retail price is approximately $80, but has higher gain (50 dB), lower noise (<1.5 dB), better multipath suppression and a more stable mean phase center. The antenna was courteously provided by Stefano Caldera of G-Red.

NEO-7P is a multiconstellation GNSS receiver: however, just one constellation at a time can be selected for a session of registration. Clearly, we chose GPS, that is the most complete and larger constellation in orbit. Each NEO-7P was connected to a laptop which ensured the power supply and allowed us to access the receiver and to configure it.

The roof that hosted the slide is surrounded by hills that partly obstruct signals for very low elevations (below 5°). Moreover, a metallic structure is near the rover location, which could cause some multipath interference. This does not constitute a limit: on the contrary, the environmental conditions are consistent with typical applications, more than the ideal test in Milano. In addition, previous experiments with geodetic receivers in this site already provided completely satisfactory results [17].

During the field work, the position of the rover antenna was gradually moved by increments of 5 mm. Horizontal (slide *X* axis) and vertical (*Y* axis) movements alternated. At least 2 h were given between consecutive movements: in this way, each horizontal/vertical position was occupied for at least 4 h. Due to the orientation of the sliding direction every 5 mm horizontal movement should theoretically translate into equal displacements in the east and north directions:

(2) δE=δN=5 mm2

Clearly, the typical pattern in time of local deformation phenomena does not follow such a regular stepwise behavior: our test is only a discrete simulation of displacements aimed at assessing the reliability of the system to identify them. By the end of the experiment, the position of the antenna was moved 10 cm in the horizontal plane and 10 cm in the vertical with respect to the initial (zero) position. During the nights no movements were applied: about 12 hourly sessions correspond to each night position but for homogeneity with other positions, only the first two hours were used and processed.

The zero position of the rover antenna was estimated as follows: two sessions of 24 h with the antenna in the zero position were acquired: the former at the beginning and the latter at the end of the experiment. The two sessions provided results consistent at 2 mm level in horizontal plane and 6 mm in height: the reference zero position was computed by averaging the two results.

### 4.2. Analysis of the Time Series between Como EPN and Rover

**Figure 5:**
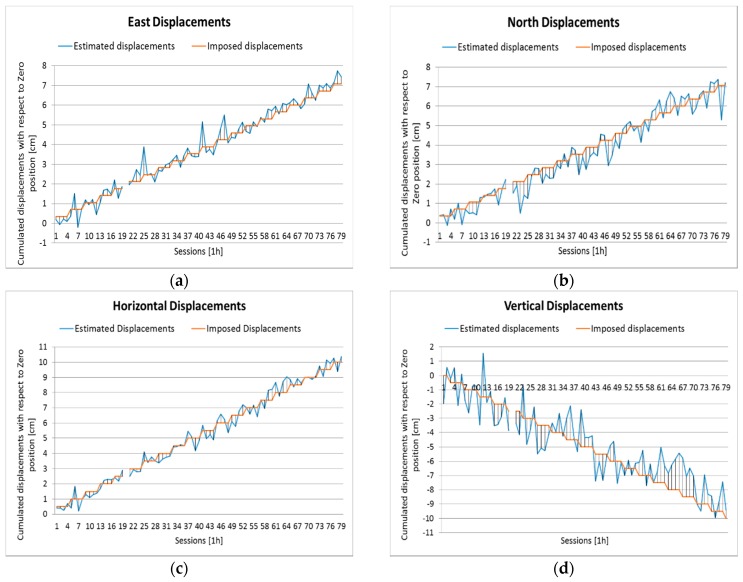
Baseline between the primary reference and rover. Hourly sessions: comparison between imposed and estimated displacements. (**a**) East; (**b**) north; (**c**) horizontal; and (**d**) up.

The Milano test confirmed the complete consistency of BSW5.2 and LGO results: because of this we adopted LGO to process the Como test. The hourly results were converted to east, north, and up displacements with respect to the zero position: they can be compared with the imposed displacements (Figure 5) and the residuals (differences between estimated and imposed displacements) can be computed. One session provided a float solution with residuals of 7 cm in east, 4.4 cm in north, and 9.1 cm in up directions: thus, this solution was removed from the list of results before proceeding to the next step of the analysis.

The estimated horizontal displacements seem quite consistent with the imposed ones, while the vertical results are significantly worse and just follow the general trend. North results are slightly worse than east results. The statistics of the residuals are presented in Table 7: means range between 1 mm (east) and 3 mm (up), while RMSEs range between 4 mm (east) and 13 mm (up); they are satisfactory but worse than those of the Milano test: this is due to worse observation conditions.

The following tests involve significance and congruence analyses. To perform them, displacements are estimated by differencing couples of estimated coordinates of the rover:

(3) $$\delta x_{ij}=x_{j}-x_{i}$$

#### 4.2.1. Significance Analysis

The significance analysis aims at assessing how many imposed displacements are identified by a significance test run on the estimated displacements. The Fisher test is adopted [33]. Given two sessions *i* and *j*, the null hypothesis is:

(4) $$H_{0}:x_{i}=x_{j}$$

If $H_{0}$ is true, then:

(5) $$\frac{\delta x_{ij}^{T}C_{\delta \delta_{ij}}^{-1}\delta x_{ij}}{m}=F_{\exp}∼F_{m,r_{i}+r_{j}}$$

where $C_{\delta \delta_{ij}}$ is the covariance matrix of the differences, $r_{i}$ and $r_{j}$ are the redundancies of the two data adjustments, and *m* is the dimension of the coordinates vector.

A threshold $F_{t}(\alpha )$ is computed for the Fisher function, such that $P(F_{m,r_{i}+r_{j}}>F_{t}(\alpha ))<\alpha$. If $F_{\exp}\leq F_{t}$ the null hypothesis is accepted as true. In the contrary case, the null hypothesis is not accepted.

In our test, α was put equal to 0.05. Let *i* and *j* be two sessions, let *n* be the number of movements between *i* and *j*. Firstly, the test is performed for the horizontal (2D, *m* = 2) and vertical displacements (*m* = 1): in these cases, *i* and *j* are separated by imposed displacements of n×5 mm. Then, the test is performed for the three dimensional displacements (3D, *m* = 3): in this case, the imposed displacement between *i* and *j* is $n\times \sqrt{2}\times 5\;\mathrm{mm}$ (the composition of *n* horizontal and vertical displacements of 5 mm). The test was applied to couples of imposed displacements up to n=5 movements.

In Table 8 the percentages of right answers for the significance test are shown. In case of 0 movement, *H*_0_ hypothesis should be accepted (“no displacement”); for other movements *H*_0_ should be rejected (“yes displacement”). In horizontal, after three movements the majority of displacements is identified: after five movements, the displacement is identified almost in the whole dataset. Similar statistics are provided by the analysis of three dimensional displacements: in this case 3 and 5 movements correspond to displacements of about 2.1 cm and 3.5 cm, respectively. As expected, vertical results are worse. Firstly, this is due to the worse accuracy of GNSS vertical estimates; moreover, the significance test in Equation (4) strengthens at the increase of *m*, therefore, the displacement test on one component is intrinsically weaker than the 2D and 3D cases.

#### 4.2.2. Congruence Analysis

The congruence analysis verifies how many estimated displacements between couples of sessions are congruent with the imposed ones. The approach explained in [18] is used here to define classification errors $\varepsilon_{\omega }$ of estimated displacements. Let $\omega_{k}$ define the class corresponding to imposed displacement ${\bar{\delta x}}_{k}=k\times 5\;\mathrm{mm},\;k=0,\ldots ,N_{max}$. Let us suppose that the probability distribution of estimated $\delta x_{ij}$ conditioned to class $\omega_{k}$ is normal with constant average ${\bar{\delta x}}_{k}$ and covariance C: a Bayesian approach is straightforward to classify each estimated $\delta x_{ij}$ in the most probable class $\omega_{k}$.

Step 1. The conditional probabilities $P(\omega_{k}/\delta x_{ij})$ are computed for each class $\omega_{k}$;
Step 2. $\delta x_{ij}$ is attributed to the class $\omega_{k}$ with the maximum $P(\omega_{k}/\delta x_{ij})$; and
Step 3. The class error is computed according to:
  (6) $$\varepsilon_{\omega }=abs(\omega_{k}-{\bar{\omega }}_{ij})$$
  where ${\bar{\omega }}_{ij}$ is the true class corresponding to the imposed ${\bar{\delta x}}_{ij}$.

Congruence analysis is performed on all the possible combinations of the 74 sessions: in total, 2775 differences, classifications, and their errors are available.

East, north, vertical, and 2D horizontal displacements are tested. A class error equal to 0 means no classification error. A class error *n* means a classification error equal to:

- $\varepsilon =n\times 5/\sqrt{2}\;\mathrm{mm}$ for east and north, and
- ε=n×5 mm for 2D and up.

**Figure 6:**
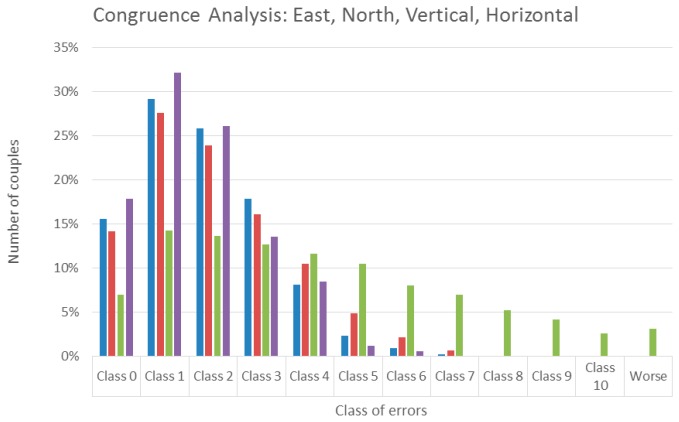
Baseline between the primary reference and the rover. Congruence analysis on estimated displacements in east, north, up and horizontal 2D directions. Percentages are of couples for each class of errors.

Figure 6 provides the detail of the results. In Table 9 the cumulated percentages are shown. The congruence analysis in the east direction provides rather satisfactory results: 66% of the couples are in class 0–2, while the maximum class error is 8 for just one result; north results are still satisfactory, but slightly worse. The 2D analysis performs better than individual east and north results. Vertical results are worse, with a significant percentage of class errors (40%) exceeding class 4.

### 4.3. Baseline between u-Blox Reference and u-Blox Rover

Then, the Slide u-blox data were processed with respect to the secondary reference u-blox: The processing and the results analysis scheme were the same as in the previous case. A first screening identifies three float sessions that are removed from the dataset.

**Figure 7:**
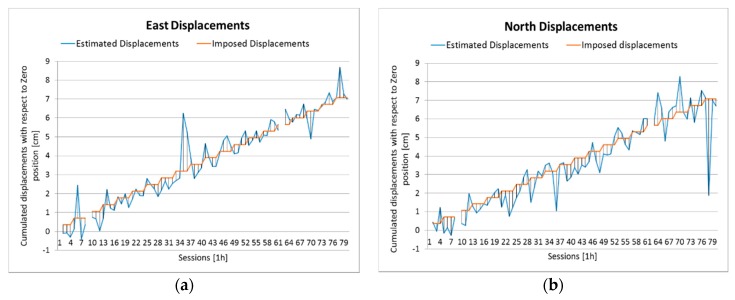

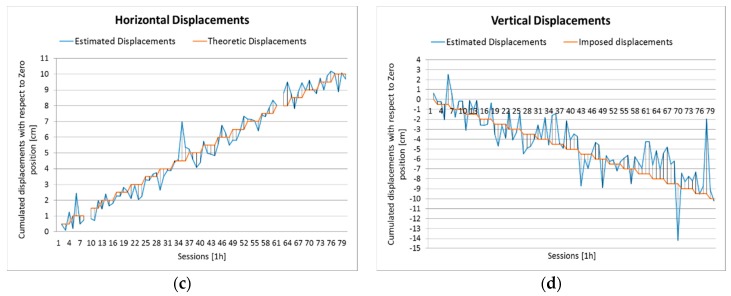
Baseline between the secondary reference and the rover. Hourly sessions. Comparison between imposed and estimated displacements. (**a**) East; (**b**) north; (**c**) horizontal; and (**d**) up.

**Figure 8:**
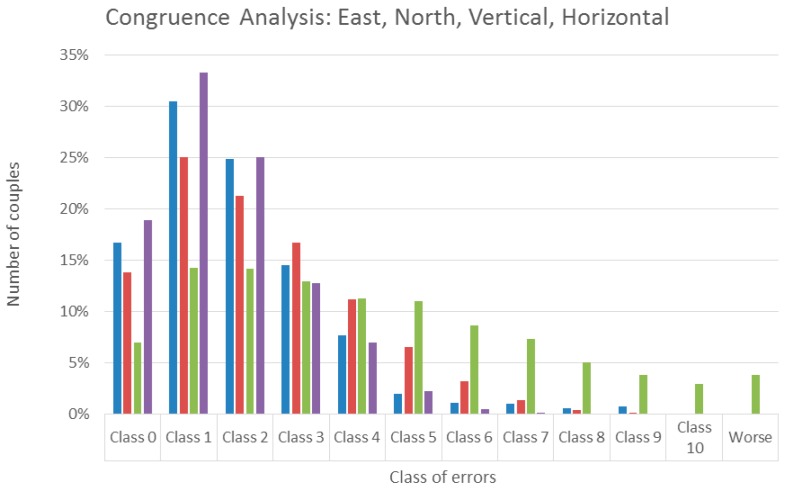
Baseline between the the secondary reference and rover. Congruence analysis on estimated displacements in east, north, up, and horizontal 2D directions. Percentages are of couples for each class of errors.

The time series of estimated displacements of east, north, and up are in Figure 7, the statistics of the residuals are presented in Table 10. Only the up mean is significant, the RMSE range between 7 mm (east) and 19 mm (up). In the significance test (Table 11), four movements are needed to detect most of the displacements in 2D and 3D. Therefore, the statistics are worse than the previous (baseline with Como EPN): this was expected, considering that in this case low-cost receivers are used both for reference and rover stations. In any case, they are still satisfactory. The results of the congruence test are in Figure 8 and Table 12: they are almost identical to those discussed in Section 4.2.

## 5. Future Works

Future experiments based on the methodology introduced by this study can be planned, in order to study two points that have been opened in the presented experiment.

Several external GNSS antennas, like the Tallysman that we used as a secondary reference, exist that have a low-cost but, in principle, should work better than the default u-blox, at least accordingly to the declared electronic characteristics. In principle, these could suffer lower data noise and multipath interference and, therefore, the accuracy in the baseline estimates could improve, especially for short sessions. In the future, an extensive comparison between different pairings of low-cost receivers and antennas will be planned.

In monitoring of vertical movements, a good accuracy of height estimates is needed: for geodetic antennas, the availability of PCV contributes significantly, especially for short sessions. In principle, PCV could improve also the performance of low-cost antennas: an experiment to estimate them, at least for the most important models, will be set up and the results will be evaluated.

## 6. Conclusions

In local monitoring, for example, a landslide, geodetic GNSS receivers are usually adopted: they provide very accurate and near-real-time results. However, geodetic instrumentation is very expensive: the initial cost, and the risk of damages and losses can discourage this approach. The objective of this research was to assess the accuracy and reliability of a monitoring system based on low-cost GNSS receivers. For the testing, two experiments were planned and performed.

Firstly (Milano Test), we analyzed a week of acquisitions on a single baseline between a geodetic reference station and a LEA-4T u-blox. This test provided a first accuracy assessment and the comparison between Bernese GPS SW and LGO package. Out of 168 results, five and four outliers were present in the results, respectively for BSW and LGO. The other hourly sessions provided accuracies well below the cm level, even for the vertical component. The two packages provided similar results.

Then (Como Test), a simulation of a local network for displacements monitoring was set up: the experiment involved three receivers. Two receivers (a geodetic receiver and a NEO-7P u-blox) acted as reference stations, another u-blox acted as rover: it was installed on a device that allowed to move its antenna by known displacements in horizontal and in vertical directions. Starting from a zero position, the rover moved 10 cm by steps of 5 mm in both directions. Each position was occupied for (at least) two hours. In total, the experiment lasted about 15 days.

In this case, all of the processing was performed by LGO. Firstly, the baseline between the geodetic reference and the rover was processed by hourly sessions. The estimated displacements were compared with the imposed ones. In this test, just one blundered session, with float results, was excluded. The other hourly sessions presented residuals smaller than 10 cm and provided accuracies at the sub-cm level in horizontal and at the cm level in vertical. They are worse than those of Milano: indeed, while Milano site presents ideal environmental conditions, Como site is more representative of typical monitoring sites, with some obstructions around. In this regard, the results are completely satisfactory.

Moreover, significance and congruence tests were applied to Como time series, in order to investigate the capability of the system to:

(1) correctly alarm in case a displacement occurred, without false alarms (significance); and
(2) correctly identify the imposed displacements (congruence).

Horizontal results are completely satisfactory: the system is able to detect displacements after three movements (15 mm) in most of the cases and the classification errors are almost always within three classes. Vertical tests are weaker: after five displacements (25 mm), only 38% of right answers are given and classification errors exceed five classes in many cases.

Then, the same approach was applied to process and analyze the baseline between the two u-blox. The results are slightly worse, but not significantly, and confirm previous conclusions.

Therefore, we can state that movements with a dominant horizontal component can be detected by local networks of low-cost GNSS receivers with sub-cm accuracy. The data processing can be performed by simple, user-friendly software and the results can be obtained by processing hourly sessions: therefore, they can provide a quasi-real-time monitoring of the phenomenon. In particular, we experimentally tested that these accuracies can be achieved even in a configuration entirely based on low-cost receivers.

## Figures and Tables

**Table 1:** BSW5.2 and LGO processing parameters (only main parameters are shown).

Parameter	BSW5.2	LGO
Observables	GPS L1 phase observations	GPS L1 phase observations
Differencing level	double	double
Session length	1 h	1 h
Troposphere	Saastamoinen	Saastamoinen
Elevation cut-off	10°	10°
Ambiguity resolution	SIGMA	yes

**Table 2:** Milano test. Three dimensional (3D) BSW5.2 residuals of blundered hourly sessions.

Session	3D (cm)
22	24
24	44
30	9
40	19
142	31

**Table 3:** Milano test. BSW5.2 hourly residuals of final solutions. E: east, N: north, and U: up.

Statistics	E (mm)	N (mm)	U (mm)
Mean	1.0	−1.5	0.8
RMSE	2.1	4.9	4.5
Min	−3.1	−10.8	−10
Max	16.8	47.5	14.3

**Table 4:** Milano test. 3D LGO residuals of blundered hourly sessions.

Session	3D (cm)
34	37
58	12
106	130
160	39

**Table 5:** Milano test. LGO hourly residuals. E: east, N: north, and U: up.

Statistics	E (mm)	N (mm)	U (mm)
Mean	0.2	−1.3	1.9
RMSE	1.8	3.7	4.4
Min	−4	−36.30	−10.2
Max	4.1	3.3	14.5

**Table 6:** Milano test. Classification of hourly residuals by percentages. E: east, N: north, and U: up.

Absolute Error Class	BSW5.2			LGO		
	E (%)	N (%)	U (%)	E (%)	N (%)	U (%)
0–5 mm	99.4	84.9	74.2	100	93.9	69.7
5–15 mm	0	13.9	25.8	0	5.5	30.3
15–30 mm	0.6 *	0.6 *	0	0	0	0
30–50 mm	0	0.6 *	0	0	0.6 *	0

Note: * represents one session.

**Table 7:** Baseline between the primary reference and the rover. Hourly sessions: mean and RMSE of the residuals. E: east, N: north, and U: up.

	E (mm)	N (mm)	U (mm)
Mean	1	−2	3
RMSE	4	6	13
Min	−9	−18	−30
Max	14	11	20

**Table 8:** Baseline between the primary reference and the rover. Significance analysis on estimated displacements and percentages of right answers to the test. Zero movement: right answer “no displacement”. Other movements: right answer “yes displacement”.

Movements between Couples	2D (%)	U (%)	3D (%)
0	78	85	80
1	20	14	28
2	29	17	46
3	63	28	68
4	88	31	90
5	99	38	100

**Table 9:** Baseline between the primary reference and the rover. Congruence analysis on estimated displacements. Cumulated percentages of classes of errors. E: east, N: north, 2D: horizontal, and U: up.

Class Error	E (%)	N (%)	2D (%)	U (%)
0	16	14	18	7
1	45	42	50	21
2	71	66	76	35
3	89	82	90	48
4	97	92	98	60

**Table 10:** Baseline between the secondary reference and the rover. Hourly sessions: mean and RMSE of the residuals. E: east, N: north, U: up.

Point ID	E (mm)	N (mm)	U (mm)
Mean	0	−2	−6
RMSE	7	9	19
Min	−15	−52	−75
Max	30	30	57

**Table 11:** Baseline between the secondary reference and the rover and significance analysis on estimated displacements. Percentages of right answers to the test. Zero movement: right answer “no displacement”. Other movements: right answer “displacement”.

Movements between Couples	2D (%)	U (%)	3D (%)
0	88	84	92
1	14	14	14
2	23	18	21
3	31	27	31
4	64	33	61
5	84	36	88

**Table 12:** Baseline between the secondary reference and the rover. Congruence analysis on estimated displacements. Cumulated percentages of classes of errors are shown. E: east. N: north. 2D: horizontal. U: up.

Class Error	E (%)	N (%)	2D (%)	U (%)
0	17	14	19	7
1	48	39	52	21
2	73	60	77	35
3	88	77	90	48
4	96	88	97	59

## Abbreviations

The following abbreviations are used in this manuscript:

GNSS	Global Navigation Satellite System
GPS	Global Positioning System
TCXO	Temperature Compensated Crystal Oscillator
PCO	Phase Center Offset
PCV	Phase Center Variations
SNR	Signal to Noise Ratio
SBAS	Satellite-Based Augmentation System
PPP	Precise Point Positioning
RTCM	Radio Technical Commission for Maritime Service
QZSS	Quasi-Zenith Satellite System
EUREF	European Reference Frame
RINEX	Receiver Independent Exchange format
LGO	Leica Geo Office
BSW	Bernese GPS Software
RMSE	Root Mean Square Error
